# Reflections on Norheim (2018), Disease Control Priorities Third Edition Is Published: Comment on "Disease Control Priorities Third Edition Is Published: A Theory of Change Is Needed for Translating Evidence to Health Policy"

**DOI:** 10.15171/ijhpm.2019.09

**Published:** 2019-02-27

**Authors:** Rachel Nugent

**Affiliations:** RTI International, Seattle, WA, USA.

**Keywords:** Priority-Setting, Cost-Effectiveness, Health Economics

## Abstract

The publication of *Disease Control Priorities*, 3rd edition (DCP3) is a major milestone in the global health world. DCP3 reviews and summarizes high quality health intervention effectiveness and cost-effectiveness evidence relevant to low- and middle-income countries and is freely available to users. This Commentary summarizes Norheim’s (2018) assessment of DCP3’s role in country health priority-setting and offers reflections on what DCP3 can continue to offer countries seeking to improve their purchasing of health.

The publication of *Disease Control Priorities*, third edition (DCP3) is a major milestone in the global health world. DCP3 reviews and summarizes high quality health intervention effectiveness and cost-effectiveness evidence relevant to low- and middle-income countries and is freely available to users. The primary intended users are finance officials who decide how much to spend on health in a country and similar decision-makers in global health institutions. In that regard the DCP3 follows the tradition of the DCP1^1^ and DCP2^2^ of addressing itself directly to the holders of health sector purse strings. The 9-volume, 7-year effort has the capacity to influence people’s health and longevity for years to come if the guidance therein is applied in many countries.

It is unusual in global health to have a communication channel to the finance officials and that is one of the main values of DCP to the health community. Typically, priority-setting in global health is influenced by medical and public health voices, accompanied by well-organized and vocal advocacy campaigns [see eg, references 10-12 in Norheim 2018]. The priority-setting process that emerges may not result in careful, rational, and pluralistic deliberation using evidence.^3^ The DCP Series, on the other hand, is all about evidence. It offers a thorough summation and evaluation of the evidence for health policies and interventions available from peer-reviewed literature, along with expert judgment from hundreds of authors from many countries. At its essence, it aims to show decision makers how to spend money wisely using the common metric of cost-effectiveness (and its variant forms of economic evaluation) interpreted through the lens of many authors’ experience. The motivating belief of the DCP Series is that this approach – when applied across different health needs – will buy more health compared to allocating resources by listening only to the voices of interest communities.

In laying out a recipe for national level health priority-setting, Norheim^4^ casts some doubt on the DCP formula. First, he says that evidence is a starting point, not an end in itself. In Norheim’s ideal system – which he describes in detail along with a theory of change – evidence “plays a key role,” but is only one input in a priority setting model. Priority setting works best if economic evidence is subsumed within a system that includes multiple steps and actors. Second, by my read Norheim would wrest control of the resource allocation decision-making from the finance ministry and return it to the health bureaucracy and academicians who would mediate among political and civil society constituencies. This includes ensuring “that the language [of priority-setting] can be understood by everyone” involved in all the stages, including gathering, interpreting and using the available evidence. Third, Norheim points to contextualization as an important element missing from the DCP.

Norheim cogently and convincingly presses his argument with examples from literature and experience to lay out a theory of change for health priority-setting. But when it comes to DCP’s role in that endeavor, his last point is the crux of the matter. What can a global project like DCP accomplish in laying out the evidence? There is a natural tension between evidence assembled for the purposes of international resource allocation decisions and priority-setting needs carefully set within national contexts. In this regard Norheim rightly points out that improved national priority-setting processes do not naturally follow publication of the evidence, however thorough, attractive, and available it is. There is no arguing with that. DCP provides necessary but not sufficient groundwork from which a national process can be developed in the form of credible authoritative external evidence but its usefulness pre-supposes existing national conditions that are favorable to national priority-setting. Norheim names three other elements of good national priority setting: (*a*) clear priorities; (*b*) publicity; and (*c*) institutionalization. All of these need to occur *within* countries, a territory that DCP has relatively little sway over.

So how can one usefully apply DCP to further those three elements? Norheim says, and I agree, that “better national priority setting goes beyond what DCP can achieve,” but this begs the question of what DCP can and should achieve.

Here is my list, based on toiling in the priority-setting factories of DCP2 and DCP3 for a significant span of my career. A DCP enterprise *should*:

- Produce a definitive state-of-the-evidence summary that is credible to specialists but not overly technical.
- Serve as a reference guide to global health evidence for a wide range of users across broad geographies.
- Employ a transparent and scientifically balanced process to reach conclusions about the evidence.
- Indicate honestly the limits of applicability and generalizability of the work’s conclusions.

Beyond those criteria, there are other contributions that a DCP can make. Norheim points to a series of steps that – over time – will create a fully nationally-owned process for health priority-setting. It includes the use of a DCP set of evidence as well as assistance from DCP producers in creating local institutions that support and maintain local expertise and introduction of evidence-based priority-setting within the national planning processes. While I agree with Norheim, I will go farther to say that DCP *could also*:

- Help evaluate locally-produced evidence and continue to strengthen national capacity, with the aim of generating new cadres of experienced analysts that will contribute to the next generation of DCP.
More assiduously examine the quality of evidence that goes into DCP and explain deviations from the norm that inform generalizability.
- Use the synthesis of evidence to propose benchmarks that can guide countries in achieving adequate and efficient spending on health.
- Create more demand for priority-setting evidence within global institutions that national health authorities engage with. Here I am thinking about donor organizations such as the Global Fund and the Bill & Melinda Gates Foundation, as well as policy advocacy groups in global health. Both types of organizations heavily influence – and sometimes distort – allocational decisions and would do well to employ solid evidence in systematic ways in so doing.
- Cultivate enduring linkages to the priorities of global agencies, professional societies, and agencies – in addition to the natural connection to the academy where most of the authors are housed.
- Finally, and perhaps most importantly, DCP could work closely with countries at different points of their priority-setting journey, especially to support universal health coverage as the agreed vehicle for advancing rational healthcare at the national level.

So, how did we – the creators of DCP3 – do on those lists of should and coulds? It is not easy to score oneself and colleagues, especially with only one year of perspective on a 7-year process. From my insider vantage point I think we did pretty well on the shoulds and even moderately well on the coulds.

- DCP3 is a credible and readable summary of evidence for a vast array of global health needs.
- It was produced through a transparent and inclusive process easily available via the DCP3 website.
- It makes no pretense to be fully generalizable but strives to be an honest representation of the evidence and to clearly express the limitations.
- It has the potential to be an important reference for public health and development professionals.

The above is a satisfying list, and yet …. DCP3 is not currently used by countries that need more priority-setting. This is Norheim’s main point and a fact that was brought home to me recently in an off-hand remark at lunch by one of DCP3’s lead volume editors. This editor, an eminent Indian researcher, said no-one talks about DCP3 in his country and therefore it “had been a waste of time.” This is a harsh judgement, but a telling one. DCP3 has not yet made a dent in national priority-setting, even in this editor’s home country which has lots of resources relative to many but is grossly misdirecting how those resources are used. This DCP3 editor had not seen a way to insert DCP3 in priority-setting in his own country in the manner that Norheim lays out. This reflects poorly on the DCP3 leadership, including myself. During the process of creating DCP3, we felt that a primary outcome was to empower the community of editors and authors – hundreds of them – that researched, reflected, and wrote the volumes to find ways to use them. They clearly believed in DCP3’s usefulness for priority-setting as was evident in many planning and draft-sharing meetings and channeled into the narrative reviews that they contributed. But, generally speaking, they have not yet found the means to employ DCP3 in priority-setting in their own countries. One possibility – hat tip to an anonymous reviewer – is to better exploit the DCP’s historical roots and publishing connection to the World Bank to inform the Bank’s interactions with its client countries. Evidence from DCP could influence lending operations in ways that are less political and more evidence-based than sometimes is the case.

The DCP3 Series Editors and Secretariat also believed that a major outcome of DCP3 would emerge from this community as it put the learning and conclusions of their work into practice and shared those ideas with their national and international professional networks. And it still may if history is any guide: DCP2 retained its unofficial designation as a “bible of global health” for many years after publication in 2006, proving its longevity as a global reference guide. DCP3 is an up-to-date and expanded replacement of DCP2. However, while time may have been a friend to DCP2, it is the enemy of DCP3. The 9 volumes took 7 years to produce and were published across 3 years. It is difficult for even the most committed priority-setters to maintain attention and track the shifting outputs and timelines, waiting for their favorite topic to finally get its turn. We attempted to reduce the wait by publishing drafts of chapters on-line before volumes were completed, but this may have also reduced the impact of the volumes when they were finally available.

Further, we are not only in a post-DCP2 world. We are in a post-DCP3 world. On-line journals publish almost in real time, Twitter offers working papers and thoughtful discussion of them even faster. The multi-volume format of DCP3 was a relic by the time the first volume was published, as evidenced by how many hard copies sit on the shelf in the DCP3 Secretariat. It would be wrong to take that as a sign that DCP3 is not being used in digital format, but frank honesty is needed to support and promote its use as *a* tool (not *the* tool any longer) for priority-setting.

That returns us to Norheim’s recipe for national priority-setting processes. As he suggests and knows through experience in Ethiopia and elsewhere, establishing a mentality for priority-setting in health and implementing it can be a long slog. He lays out a timeline and confirms that it can start with DCP3. He rightly states that the evidence needs better packaging, and it will need translation based on interest and capacity, supported by political will. And it needs continued funding. Where a global project such as DCP fits into Norheim’s actions for better priority setting within a country is clear: it should provide evidence to guide countries and work with them on methods to translate that evidence and to develop the in-house capacity to produce it locally. That is the future of DCP and it is an important one.

## Ethical issues

Not applicable.

## Competing interests

Author declares that she has no competing interests.

## Author’s contribution

RN is the single author of the paper.
